# Intraspecific sequence comparisons reveal similar rates of non-collinear gene insertion in the B and D genomes of bread wheat

**DOI:** 10.1186/1471-2229-12-155

**Published:** 2012-08-30

**Authors:** Jan Bartoš, Čestmír Vlček, Frédéric Choulet, Mária Džunková, Kateřina Cviková, Jan Šafář, Hana Šimková, Jan Pačes, Hynek Strnad, Pierre Sourdille, Hélène Bergès, Federica Cattonaro, Catherine Feuillet, Jaroslav Doležel

**Affiliations:** 1 Centre of the Region Haná for Biotechnological and Agricultural Research, Institute of Experimental Botany, Sokolovská 6, Olomouc, CZ-77200, Czech Republic; 2 Institute of Molecular Genetics, Vídeňská 1083, Praha, CZ-14220, Czech Republic; 3 INRA University Blaise Pascal UMR 1095 Genetics Diversity Ecophysiology of Cereals, Clermont-Ferrand, F-63100, France; 4 INRA, National Resources Centre for Plant Genomics, Castanet Tolosan Cedex, F-31326, France; 5 Instituto di Genomica Applicata, Via J. Linussio 51, Udine, 33100, Italy

**Keywords:** Wheat, BAC sequencing, Homoeologous genomes, Gene duplication, Non-collinear genes, Allopolyploidy

## Abstract

**Background:**

Polyploidization is considered one of the main mechanisms of plant genome evolution. The presence of multiple copies of the same gene reduces selection pressure and permits sub-functionalization and neo-functionalization leading to plant diversification, adaptation and speciation. In bread wheat, polyploidization and the prevalence of transposable elements resulted in massive gene duplication and movement. As a result, the number of genes which are non-collinear to genomes of related species seems markedly increased in wheat.

**Results:**

We used new-generation sequencing (NGS) to generate sequence of a Mb-sized region from wheat chromosome arm 3DS. Sequence assembly of 24 BAC clones resulted in two scaffolds of 1,264,820 and 333,768 bases. The sequence was annotated and compared to the homoeologous region on wheat chromosome 3B and orthologous loci of *Brachypodium distachyon* and rice. Among 39 coding sequences in the 3DS scaffolds, 32 have a homoeolog on chromosome 3B. In contrast, only fifteen and fourteen orthologs were identified in the corresponding regions in rice and *Brachypodium*, respectively. Interestingly, five pseudogenes were identified among the non-collinear coding sequences at the 3B locus, while none was found at the 3DS locus.

**Conclusion:**

Direct comparison of two Mb-sized regions of the B and D genomes of bread wheat revealed similar rates of non-collinear gene insertion in both genomes with a majority of gene duplications occurring before their divergence. Relatively low proportion of pseudogenes was identified among non-collinear coding sequences. Our data suggest that the pseudogenes did not originate from insertion of non-functional copies, but were formed later during the evolution of hexaploid wheat. Some evidence was found for gene erosion along the B genome locus.

## Background

Polyploidy is considered one of the main driving forces of plant evolution and speciation. Whole genome duplication (WGD) provides a substrate for plant genome evolution, diversification and adaptation. The presence of two or more copies of the same gene reduces selection pressure and enables sub-functionalization and neo-functionalization. The analysis of whole-genome sequences revealed a frequent and often repeated occurrence of genome doubling during the evolution of higher plants. Even plants with relatively small genomes such as *Arabidopsis thalianaBrachypodium distachyon* and *Malus domestica* have experienced polyploidization events during their evolution [1-3].

Some whole-genome duplication events occurred tens or even hundreds of million years ago (MYA). These paleoploidization events are not easy to detect by cytogenetic methods, but can be identified at the sequence level. In addition to paleoploidy, some plant species such as bread wheat (*Triticum aestivum* L.) experienced genome duplication more recently. In this case, intact homoeologous chromosomes persist without apparent structural changes except for some interchromosomal rearrangements [4]. The genome of hexaploid bread wheat (2n = 6x = 42, 1C ~ 17Gb) was formed by two distinct hybridization events. The first took place 0.2 – 0.5 MYA [5] and involved a diploid wild species *T. urartu* (AA genome) and a diploid species related to *Aegilops speltoides* (SS genome). This event gave rise to the allotetraploid *T. dicoccoides*, the ancestor of the cultivated durum wheat *T. turgidum* with the AABB genome. Only about 10,000 years ago, a diploid donor of the D genome ( *Ae. tauschii*) hybridized with *T. dicoccoides* to produce the ancestor of cultivated *Triticum aestivum* L. with the AABBDD genome [6].

In addition to WGD, tandem and dispersed duplications of individual genes and gene clusters are involved in genome evolution. These events disrupt collinearity between genomes of related species and limit the potential of model species with small genomes as surrogates for gene mapping and cloning in crops with complex genomes. The mechanisms leading to gene duplication are still not well understood, but transposable elements (TEs) which account for more than 85% of the wheat genome may play a critical role. TE activity can result in gene duplication through at least two mechanisms. First, a gene can be captured by a TE and copied together with the transposable element to a new location. The ability of TEs to capture genes and gene fragments has been well documented in plants [2,7,8]. Another mechanism of gene duplication coupled with TE activity is based on double-strand break repair through synthesis-dependent strand annealing (SDSA) which can accompany TE insertion [9]. In this case the sequence fragment containing a gene is used as filler DNA to repair the double strand-break, which occurs during filling of target site duplications. TE-mediated gene movements result in the insertion of genes at non collinear positions. Recent studies indicate that collinearity of wheat chromosomes to syntenic regions in two related grass species *Brachypodium* and rice is lower than estimated previously [10]. It has been suggested that the higher number of non-collinear genes results from TE activity [10,11]. However, Choulet *et al.*[10] and Wicker *et al.*[11] differ in the estimated rate of non-collinear gene insertion. While Choulet *et al.*[10] suggest the same fraction of non-collinear genes in the three genomes of bread wheat and in barley, Wicker *et al.*[11] found significant differences in the gene insertion rate among the wheat group 1 chromosomes.

Gene loss is another mechanism leading to the observation of apparently non-collinear genes. Massive gene loss may accompany chromosome re-arrangements as documented in *Arabidopsis*[12]. The loss can be significant as shown by [13] who observed 50% gene loss from the two progenitors of maize within about 5 million years after polyploidization. In Arabidopsis, almost half of the genome was lost during the last 10 million years of evolution [12]. Loss of genes from one of the homoeologous segments was also described in more recent allotetraploids such as *Arabidopsis suecica* and *Tragopogon mirus*[14,15]. Thus, gene loss in *Brachypodium* and rice after paleopolyploidization could be responsible for the apparent increase in the number of non-collinear genes in wheat, even if the genes remain in their ancestral positions in wheat.

While previous reports identified major mechanisms driving the evolution of plant genomes, additional large-scale genomic sequences are needed to investigate non-collinear gene frequency, duplication mechanisms and non collinear gene function in more details. Due to its recent evolutionary history, hexaploid wheat is a good model to study these processes. As the sequencing of the wheat genome has been hampered by polyploidy and genome size (17Gb), the international wheat genome sequencing consortium (IWGSC) has adopted a chromosome based approach that relies on the construction of physical maps from isolated chromosomes and chromosome arms [16]. In wheat, chromosome arms represent 1.3 to 3.4% of the whole genome and thus chromosome genomics offers a great reduction in sample complexity. The methods for chromosome isolation and flow cytometric sorting are well established in wheat [17,18] and BAC libraries, which are being constructed from all chromosome arms [19], were shown suitable to construct sequence-ready physical maps [20].

The aim of the study was to examine whether homoeologous genomes of hexaploid wheat are characterized by the same rate of non-collinear gene insertion and if orthologous genes retain the same activity. We used the available chromosome arm-specific BAC library of bread wheat and sequenced a locus on chromosome 3D orthologous to contig ctg0954b on chromosome 3B. Contig ctg0954b (~ 3.1 Mb) contained 53 coding sequences and displayed high degree of non-collinearity with *Brachypodium* and rice (62% of CDS are non-collinear) [10]. Due to its character, the locus appears a suitable candidate for comparison of non-collinear gene insertions among homoeologous genomes of hexaploid wheat. We have identified and sequenced 1.6 Mb region on chromosome arm 3DS corresponding to part of the contig ctg0954b and compared gene content, gene by gene. This first ever comparison of Mb-sized orthologous regions of hexaploid wheat revealed similar rates of non-collinear gene insertion in the B- and D-genome loci. About two thirds of gene insertions are shared between the two loci, while the remaining gene movements took place after the divergence from common ancestor. Moreover, our results indicate a reduction of gene expression along the B locus as compared to the D locus.

## Methods

### Selection of BAC clones for sequencing

Minimum Tiling Path (MTP; 3,827 clones ordered in ten 384-well plates) of the physical map of wheat chromosome arm 3DS was screened using PCR to select BAC clones homologous to 3B contig ctg0954b. Three-dimensional pools of MTP were prepared so that ten plate pools represent individual plates, 16 row pools consist of clones from particular row of all ten plates, and 24 column pools consist of clones from particular columns of the ten plates. BAC clones of each pool were transferred using GeneTAC G^3^ robotic workstation (Genomic Solutions) onto solid 2YT medium and incubated for 16 hours at 37°C. Bacterial colonies were then washed to liquid 2YT medium and incubated for further 8 hours at 250 rpm and 37°C. Bacterial DNA was isolated by standard alkaline lysis with minor modifications. PCR reaction contained 1× PCR buffer, 0.2 mM each of dNTPs, 0.5 U Taq polymerase, 0.01% Cresol Red, 1.5% sucrose, 20 ng DNA of particular pool and 1 μM primers (for primer list see Additional file 1: Table S1). PCR was performed in a C1000 Touch Thermal Cycler (Bio-Rad) as follows: initial denaturation at 95°C for 5 min; 35 cycles of 95°C for 30 sec, 60°C for 30 sec and 72°C for 30 sec; final extension at 72°C for 5 min. PCR product were separated on 1.5% agarose gel, stained by ethidium bromide and visualised on IN Genius Syngene Bio Imaging system (Syngene). BAC addresses of positive clones were de-convoluted from positive pools using Elephant software [21]. A set of 24 BAC clones covering the region corresponding to ctg0954 was selected for sequencing.

### Sequencing BAC clones

DNA from the 24 BAC clones homoeologous to 3B contig ctg0954b was isolated using adapted Midi-prep protocol [22]. All clones were grown and isolated separately. They were incubated overnight at 37°C in 10 ml of 2YT medium and isolated by standard alkaline lysis procedure [22]. The isolation yielded on average 700 ng DNA. To prepare paired-end libraries, aliquots of all clones were pooled after DNA isolation. The amount of pooled DNA was 600 and 200 ng per BAC clone for preparation of 8-kb and 3-kb pair-end libraries, respectively. In total, 24 unpaired libraries were created for individual clones, 3-kb and 8-kb paired-end (PE) libraries were created from pooled DNA representing all 24 BAC clones. Each library was individually processed using 454/Roche Titanium shotgun or standard paired-end sequencing kits and sequenced on 454/Roche GS FLX sequencer according to manufacturer’s specifications. Raw sequence data were deposited in Sequence Read Archive (study accession number: ERP001545).

### Sequence assembly

Newbler assembler (version 2.0) was used for automated assembly. First, unpaired reads of individual BAC clones were assembled using algorithm optimised for large complex genomes and following (default) settings: Minimum read length – 20 bp; Seed step – 12; Seed length – 16; Seed count – 1; Minimum overlap length – 40; Minimum overlap identity – 90%; Alignment identity score – 2; and Alignment difference score – minus 3. These assemblies resulted in unordered contigs. 3-kb and 8-kb pair-end reads of pooled clones were assembled together using the same assembling procedure as for individual BAC clones producing contigs ordered in scaffolds. Following this, the assembly was manually edited. Full sequencing reads (not clipped by assembler) at the end of individual scaffolds were compared to small repetitive contigs using Blastn (Minimal identity: 99%; and Minimal alignment length: 100 bp). Whenever two scaffolds matched ends of the same contig, they were merged and gap was closed using a contig sequence. Gaps in the scaffolds were closed in a similar way. All the manual editing was done using Geneskipper (EMBL, Heidelberg). The sequences of individual BAC clones were selected from scaffolds through alignment of Sanger generated BAC-end sequences (Bartoš *et al.*, unpublished) with scaffolds using Blastn.

### Sequence annotation

The semi-automated pipeline TriAnnot was used for sequence annotation [23] and the annotation was curated under Artemis v.11 [24]. BAC clones were annotated individually and then merged to produce annotations of two large scaffolds. Sequence of BAC clones was first compared using RepeatMasker (http://repeatmasker.org) to database of repetitive elements build in house from TREP (http://wheat.pw.usda.gov/ITMI/Repeats/) and from repeats on wheat chromosome 3B [10]. RepeatMasker-based annotations of transposable elements were then curated using Dotter[25] to detect the exact borders of elements when possible. When target site duplications (TSDs) and long terminal repeats (LTRs) or terminal inverted repeats (TIRs) were identified, the element was considered 'complete'. Finally, all repetitive elements were classified according to criteria described in Choulet *et al.*[10] and following the nomenclature of Wicker *et al.*[26]*.*

Similarity searches were performed with Blast[27] against *Brachypodium distachyon* and *Oryza sativa* annotated genes and proteins, ESTs and Unigenes available for *Triticum aestivumHordeum vulgare* and all other Poaceae, and finally, against full-length cDNAs available for wheat and barley. For similarity search against transcripts, significant Blastn hits were then aligned precisely with Gmap[28] to identify splice sites. The results of all analyses were written as sequence features in EMBL format and combined under Artemis to design most probable gene models. Gene models displaying premature stop codons, frameshift mutations or deletions up to 30% of complete homologue in databases were considered as pseudogenes.

### Sequence comparison to orthologous loci in *Brachypodium* and rice and on chromosome 3B

We used NCBI Blast[27] with default settings, except the E-value, which was set to 1e^-10^, for all homology searches among 3DS and 3B gene sets and rice and *Brachypodium* coding sequences. First, coding sequences of all (pseudo)genes on 3DS locus were compared by Blastn to gene set from the 3B contig ctg0954b [GenBank:FN564434] [10] and 3B-specific homologues were identified. Then, 3DS genes with no homologue identified among 3B genes were compared to complete sequence of 3B contig ctg0954b to resolve inconsistencies in annotations between the two loci. In a similar way, 3B genes with no homologue among 3DS genes were searched against complete 3DS locus. Both gene set were then compared to coding sequences in *Brachypodium* (Bradi1.0 gene set; http://www.brachypodium.org/) and rice (RGAP6.1 gene set) [29] to identify best homologues in *Brachypodium* and rice genome. For detailed comparison of gene – pseudogene pairs, a sequence region spanning a gene plus 1 kb upstream and downstream sequence was selected from the 3DS locus. Corresponding sequence containing pseudogene was selected from the 3B locus. The comparison was done using Artemis Comparison Tool[30].

### Promoter and expression analysis

We extracted 1 kb sequence upstream to start codon for each coding sequence found at 3DS- and 3B-specific loci. We predicted polymerase II promoters, transcription start sites and TATA-box positions using TSSP [31] at http://linux1.softberry.com/berry.phtml. 1,071,335 ESTs publicly available in GenBank (http://www.ncbi.nlm.nih.gov/genbank/) were used for expression analysis of CDS. Standalone Blastn programme was used to perform a similarity search with following parameters: reward for a nucleotide match 2; expectation value 1e^-10^; and default settings for all other parameters. Only blast hits with 100% identity and minimal length of 100 bp were considered.

## Results and discussion

### Selection of BAC clones for sequencing

A Minimum Tiling Path (MTP) was established during the construction of physical map of chromosome arm 3DS (Bartoš *et al.*, unpublished) using the TaaCsp3DShA 3DS-specific BAC library [19]. Three-dimensional pools of the MTP comprising 3,827 clones were prepared and screened using twelve markers derived from ESTs (see Additional file 1: Table S1) that were mapped previously to a completely sequenced and annotated 3 Mb region of chromosome 3B (ctg0954b, [GenBank:FN564434]) [10]. Four markers identified single clones each, while the remaining markers identified two clones each. In five cases, two positive BAC clones belonged to different contigs on 3DS, indicating contig overlap. In one case, the overlap was supported by three molecular markers. In total, three overlaps between contigs on 3DS were identified. All these overlaps were further confirmed by sequence assembly. In total, eight contigs homologous to 3B contig ctg0954b (before contig merge) were identified. The MTP of these contigs consisted of 24 BAC clones (Table 1) that were sequenced by 454 GSFX titanium technology.

**Table 1:** Distribution of BAC clones in physical map contigs and positions of positive markers

**Clone name**	**Original contig**	**Matching EST**		
**Scaffold A (ctg447)**				
TaaCsp3DShA_0045N17	ctg447			
TaaCsp3DShA_0065O14	ctg447			
TaaCsp3DShA_0008G17	ctg447	BE412128		
TaaCsp3DShA_0023I21	ctg447			
TaaCsp3DShA_0072N17	ctg447			
TaaCsp3DShA_0082H07	ctg626			
TaaCsp3DShA_0072H10	ctg626	BE606515		
TaaCsp3DShA_0037C23	ctg626	BE606515		
TaaCsp3DShA_0031N24	ctg1091	BE499148		
TaaCsp3DShA_0009G11	ctg1091	BE499148	BE427255	
TaaCsp3DShA_0004D04	ctg1091		BE427255	
TaaCsp3DShA_0087O11	ctg801	BJ303565	TC57899	TC57902
TaaCsp3DShA_0058C08	ctg432	BJ303565	TC57899	TC57902
TaaCsp3DShA_0024N06	ctg432			
TaaCsp3DShA_0011A13	ctg432	BF484268	BE398286	
TaaCsp3DShA_0055O10	ctg456	BF484268	TC268917	
TaaCsp3DShA_0015G21	ctg456			
TaaCsp3DShA_0036F12	ctg456			
**Scaffold B (ctg1484)**				
TaaCsp3DShA_0052A05	ctg1484	BJ227880		
TaaCsp3DShA_0054D16	ctg2749			
TaaCsp3DShA_0053N07	ctg2749	BJ227880	BJ222861	
TaaCsp3DShA_0089C07	ctg2749			
TaaCsp3DShA_0090J06	ctg2749			
**Singleton**				
TaaCsp3DShA_61C24	ctg801			

### Shotgun BAC sequencing and assembly

454 unpaired reads were generated from each of the 24 BACs. This produced 3,418 to 46,129 high quality *E. coli* filtered reads per BAC clone. In total, 592,593 reads with an average read length of 328 bp were generated. These were assembled into 13 to 155 large contigs (>500 bases) with an average of 35 contigs per clone (Additional file 2: Table S2). The lowest number of large contigs was obtained for the assembly of BAC clone TaaCsp3DShA_0065O14 which has the smallest insert (66.8 kb compared to an average insert size of 114.8 kb for all 24 BACs; Table 2). The N50 value for the individual sequenced BAC clones ranged from 913 to 13,617 bp. The quality of the BAC sequence assemblies obtained using unpaired sequence reads was low with several assemblies resulting in more than 50 unordered contigs and N50 values that were low when compared to clone inserts. Clearly, in large and TE rich genomes like wheat, unpaired short read sequencing cannot provide high quality assemblies. Newbler assembler, which was used to produce assembly, fragmented sequence in all cases when the repetitive elements were too similar to be discriminated properly. In order to obtain a reference sequence for the 3DS contigs, we decided to sequence all BAC clones in one pool using paired-end reads.

**Table 2:** The order and positions of BAC clones in scaffolds A and B after final assembly of the sequence

	**Clone name**	**Contigs**	**Bases**	**Start**	**End**
**A**	**Scaffold A (ctg447)**	**40**	**1,264,820**		
A01	TaaCsp3DShA_0045N17	7	107,963	1	107,963
A02	TaaCsp3DShA_0065O14	3	66,793	97,146	163,938
A03	TaaCsp3DShA_0008G17	4	112,416	123,308	235,723
A04	TaaCsp3DShA_0023I21	5	138,394	208,205	346,598
A05	TaaCsp3DShA_0072N17	3	132,725	266,400	399,124
A06	TaaCsp3DShA_0082H07	1	121,720	346,593	468,312
A07	TaaCsp3DShA_0072H10	1	121,299	443,494	564,792
A08	TaaCsp3DShA_0037C23	1	123,986	460,986	584,971
A09	TaaCsp3DShA_0031N24	4	110,210	562,811	673,020
A10	TaaCsp3DShA_0009G11	2	103,381	628,179	731,559
A11	TaaCsp3DShA_0004D04	6	141,661	660,744	802,404
A12	TaaCsp3DShA_0087O11	6	126,287	731,586	857,872
A13	TaaCsp3DShA_0058C08	1	110,681	834,837	945,517
A14	TaaCsp3DShA_0024N06	4	120,824	912,828	1,033,651
A15	TaaCsp3DShA_0011A13	2	107,890	994,129	1,102,018
A16	TaaCsp3DShA_0055O10	2	122,114	1,056,627	1,178,740
A17	TaaCsp3DShA_0015G21	2	104,099	1,074,652	1,178,740
A18	TaaCsp3DShA_0036F12	15	101,762	1,163,059	1,264,820
**B**	**Scaffold B (ctg1484)**	**22**	**333,768**		
B01	TaaCsp3DShA_0052A05	12	109,012	1	109,012
B02	TaaCsp3DShA_0054D16	8	113,313	31,157	114,469
B03	TaaCsp3DShA_0053N07	5	112,439	89,026	201,464
B04	TaaCsp3DShA_0089C07	8	114,151	161,333	275,483
B05	TaaCsp3DShA_0090J06	6	114,272	219,496	333,767
**C**	**Singleton**				
C01	TaaCsp3DShA_0061C24	8	112,713	1	112,713

### Paired-end assembly of pooled BACs resulted in two scaffolds

Two paired-end sequencing runs (3-kb and 8-kb) of pooled BAC DNA resulted in a total of 686,372 high quality *E. coli* filtered reads. Assembly with Newbler produced 282 large contigs and 33 scaffolds. The largest scaffold consisted of 548,429 bases. TEs represented main problem of the assembly as in the case of shotgun assemblies. However, the use of paired-end libraries allowed to bridge problematic regions and built scaffold significantly longer than contigs of shotgun assemblies. The number of contigs in the assembly was reduced to 62 contigs in two scaffolds by manual revision and gap closing. As repetitive elements at the end of scaffold may cause misassembly during manual merging, we have compared scaffold ends against database of Triticeae repeats (http://wheat.pw.usda.gov/ITMI/Repeats/). Among 66 scaffold ends, eight have at least 100 bp hit with more than 95% identity (data not shown). Based on these results we have selected stringent criteria for end merging (see Methods). The sequences of the 24 individual BACs were extracted from the assembly based on Blastn search against available BAC end sequences (BES). As expected, many of the BAC sequences overlapped and could be ordered into 2 BAC contigs corresponding to the two scaffolds A and B, with 18 and 5 BACs, respectively (Table 2). In order to test the coverage of the locus, sequence reads were mapped to the assembled sequence. The results showed even distribution (Additional file 3: Figure S1).

One BAC (TaaCsp3DShA_0061C24) remained single without any neighbouring clones. This clone was removed from the physical map of contig ctg801 and excluded from further analysis. Moreover, sequence comparisons identified overlaps of clones that were not found during physical map assembly. Using this information, six and two physical map contigs were merged in contigs ctg447 and ctg1484, respectively thereby resulting in a physical map of 2 contigs for this region. Sequence of scaffolds A [EMBL:HE774676] and B [EMBL:HE774675] correspond to physical map contigs ctg447 and ctg1484, respectively.

Thus, the use of paired-end reads resulted in a 13-fold decrease (from 829 to 62) in the number of contigs and dramatically improved contig ordering in the 24 BACs. After consensus building, sequences of scaffolds A and B comprised 1,264,820 bases in 40 contigs and 333,768 bases in 22 contigs with a N50 of 48,251 and 22,881 bp, respectively. Four clones were assembled in a single contig and sequence of four additional clones contained only a single gap. Two thirds of all gaps (40/60) in the assembly were found at two specific repetitive regions. Twenty six gaps occurred within LTRs of retrotransposons and 14 in the protein coding part of CACTA elements. The remaining gaps most probably contained cores of GC islands, which were not sufficiently amplified in the emulsion PCR. These results confirm that paired end reads are absolutely necessary to assemble wheat genome sequences and that libraries of at least 8 kb are important to span entire TEs. Despite the fact that two thirds of sequences in the sequenced contigs were mobile DNA elements, some of them present in more than ten copies and with highly identical long terminal repeats of LTR-retrotransposons, the quality of our assembly was comparable to that of *Cucumis melo* with a repeat content of only 7.2% [32]. In a similar experiment [33] sequenced six pools consisting of 26–28 sequential BACs from physical map of *Oryza barthii*. Final assemblies of *O. barthii* pools contained nine to 31 unordered scaffolds. Our approach based on mate-pair reads resulted in the assembly of a similar number of scaffolds (33), which was further reduced to only two by manual editing.

### Locus composition reflects overall wheat D genome content

Annotation of the assembled sequences identified a total of 1,059,398 bp (66.3%) as part of repetitive landscape. Most of this space (33.4%) was occupied by Gypsy superfamily of LTR retroelements, which is the most frequent among all superfamilies identified (for details see Table 3). About 12% of the repetitive space could not be assigned to any known TE superfamily. In addition, 39 coding sequences (CDSs) covering 57,167 bp (3.6% of the assembly) were identified in the two scaffolds.

**Table 3:** Classification of repetitive elements in the assembled sequence

**Class**	**Order**	**Superfamily**	**No. elements**	**Cumulative length**
Retrotransposons			116	663,798 (62.7%)
	LTR_retrotransposons		108	647,191 (61.1%)
		Copia	46	236,440 (22.3%)
		Gypsy	50	354,331 (33.4%)
	LINE		8	16,607 (1.6%)
DNA transposons			92	267,505 (25.2%)
	TIR		90	266,656 (25.2%)
		CACTA	39	256,633 (24.2%)
		Harbinger	2	433 (0.0%)
		Mutator	4	3,609 (0.3%)
		Mariner	41	5,096 (0.5%)
Unclassified			349	128,095 (12.1%)
Total			557	1,059,398 (100%)

The estimated fraction of “known repeats” in the 3DS locus (58.3%) is in a complete agreement with the 56.8% observed after BAC end sequencing of the D genome progenitor of *Ae. tauschii*[34]. In contrast, Li *et al.*[13] reported 70.7% of known repeats in the *Ae. tauschii* genome by plasmid end sequencing. However, it is not clear, whether they counted repetitive elements in terms of nucleotides or reads, which could bias the results. Recently, You *et al.*[35] sequenced genome of *Ae. tauschii* to 1.35x coverage by 454 and estimated that repeats represented 59% of the genome. Coding sequences represented 3.6% bases in our sequence data set, a value intermediate between the 2.5% and 4.8% estimated by Li *et al.*[13] and Akhunov *et al.*[34], respectively. Thus, we conclude that the composition of the Hga 3DS locus reflects general features of the bread wheat D genome and its progenitor *Ae. tauschii*.

The average distance between genes at the Hga 3DS locus was 25.3 kb (median 17.7 kb). We estimated gene density also for the homoeologous region of chromosome 3B to compare the degree of genome expansion in intergenic regions. Assuming the same gene content in the three wheat genomes (which evolved from closely related diploid species), gene density should be lower in the B than in the D genome. As the B genome is about 27 per cent larger than the D genome [19], the median of gene distance at this particular chromosome 3B locus (21.8 kb) agrees well with the genome size ratio. However, the locus seems to be enriched for genes as compared to the whole genome as median gene distance of chromosome 3B was estimated twice larger (42.5 kb) based on analysis of 175 genes across 13 Mb-sized contigs [10] reflecting a slightly higher gene density at the telomeres compared to the centromeric regions in wheat [10,36].

### Similar rate of non-collinear gene insertion at homoeologous loci on chromosomes 3DS and 3B

**Figure 1:**
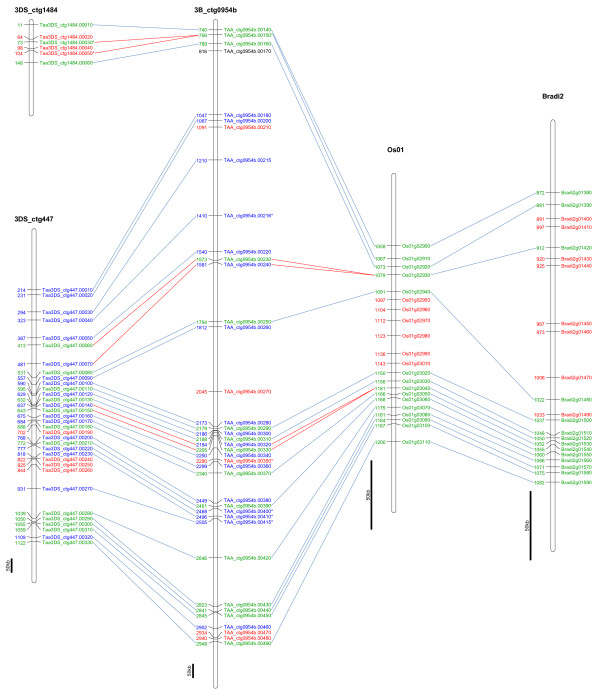
**Comparison of the gene content at the Hga locus on 3DS with its homoeologous sequence on wheat chromosome 3B and homologous regions in rice (Os01) and*Brachypodium*(Bradi2) genome.** Genes shared at the two wheat loci and at least one model genome at collinear positions are labelled in green. Genes shared between the homoeologous chromosomes 3D and 3B only are labelled in blue. Genes unique to one of the compared regions are labelled red. Pseudogenes and gene fragments are marked by an asterisk. Tandem duplications are highlighted by red lines associating both copies with particular orthologous gene. The copy with higher homology to orthologous genes is supposed to be collinear whereas less conserved gene in pair is considered non-collinear. The bar corresponds to 50 kb sequence in each locus.

A total of 38 CDSs were found at the homoeologous locus on chromosome 3B [GenBank:FN564434] [10]. Thirty two of them (84%) were found at collinear position at the 3DS locus. Five other were specific for the 3B locus. The last CDS was either be in the gap between the two 3DS contigs or missing on the 3DS locus. Since it was not possible to ascertain its presence or absence in the locus, the CDS was excluded from further comparisons. Conversely, seven CDS were only found at the 3DS locus (Figure 1). We then compared the CDS specific for one locus with the complete sequence of the other locus to reveal possible annotation discrepancies. Blastn search of CDS annotated on 3DS but not on 3B against the complete sequence of 3B ctg0954b revealed an additional gene fragment (~500 bp) homologous to Taa3DS_ctg447.00270, named in this study TAA_ctg0954b.0415. Reverse blast search of 3B specific CDS against the complete 3DS locus did not identify any additional CDS on 3DS. Moreover, two CDS (TAA_ctg0954b.00216 and TAA_ctg0954b.0217) were found to be fragments of a single CDS homologous to Taa3DS_ctg447.00040 and a name TAA_ctg0954b.00216 is used for merged CDS. After annotation revisions, 38 CDS were identified on the 3B locus.

All CDSs found at the 3DS locus were also compared by Blastn against the complete sets of gene models from rice and *Brachypodium* downloaded from ftp://ftp.plantbiology.msu.edu/pub/data/Eukaryotic_Projects/o_sativa/annotation_dbs/ (RGAP 6.1) and http://ftp.brachypodium.org/ (Bradi_1.0). The best matching gene model in each species was considered a homolog. Chromosomes syntenic to wheat group 3 in rice and *Brachypodium* are Os01 and Bradi2, respectively [2,37]. The regions corresponding to the wheat 3B and 3D sequenced loci are Bradi2g01380 – Bradi2g01590 in *Brachypodium* and Os01g02900 - Os01g03110 in rice. Twenty two and 21 genes were found in the corresponding regions in *Brachypodium* and rice, respectively (Figure 1). Only fourteen of them were found at collinear positions in all four syntenic regions (Brachypodium, rice, and wheat B and D genomes). One additional gene was found conserved in rice and the wheat B and D genomes but not *Brachypodium,* suggesting a deletion in the later species. Collinear genes accounted for 63.6% and 71.4% of the coding sequences in *Brachypodium* and rice, respectively. Overall these results confirm earlier observation that *Brachypodium* and rice are equally useful for comparative genomics applications [38]. In contrast, these collinear genes represent only 38.5% and 39.5% of the coding sequences in wheat at the 3DS and 3B loci.

Non-collinear genes in wheat were classified into two groups. The first group contained genes shared between the two wheat loci but missing at syntenic positions in *Brachypodium* and rice. Three-quarters (17) of the non-collinear genes fell into this category. The second group comprised genes found either at the 3DS (7) or the 3B (5) locus. Three and two genes arose from tandem duplication at 3DS and 3B locus, respectively. A copy of a gene with the highest sequence similarity ( Blastn) to the orthologous genes in *Brachypodium* and rice was considered collinear, whereas the less conserved gene was counted as non-collinear.

Our data are consistent with the observations that the rate of non-collinear gene insertion in the diploid wheat progenitor *Ae. tauschii*[38] and hexaploid wheat [10,11] is higher when compared to non-Triticeae grass species. However, the two studies on hexaploid wheat differed in the estimated gene insertion rate among the homoeologous genomes. The present results support the conclusions of Choulet *et al.*[10] and indicate same number of non-collinear gene insertions for the wheat B and D genomes. Our results also suggest that studies on genome evolution are more robust when performed with complete sequences rather than with survey sequences, which are by definition incomplete and can introduce bias when determining gene loss and other structural genome changes.

Wicker *at al*[11] proposed two mechanisms for gene movement/insertions: double strand repair and TE mediated transfer. The latter has been suggested also by Choulet *et al.*[10] who linked recent TE activation with the increased number of relocated genes in diploid progenitors of hexaploid wheat. Acquisition of genes/gene fragments by TEs was documented also in other grass genomes such as rice, maize, Sorghum and *Brachypodium*[7,8,39,40]. TEs with a capability to acquire gene fragments comprise Pack-MULE [7], Helitrons [8], CACTA [10,39,40], Harbinger [40], DNA transposons and LTR retrotransposons [41]. A by-product of TE activity is the expansion of target regions. However, the D genome is smaller than the B genome [19], suggesting a lower TE activity and consequently less non-collinear genes in this genome. The comparable number of non-collinear genes in the homoeologous B and D genome loci indicates either reduced efficiency of gene movement by TEs at the B locus (i.e. genes moved per TE insertion) or increased role of other mechanisms in gene movement at D genome locus. However, despite the Mbp-size, the locus analysed in the present work is relatively small and our observations need to be confirmed after comparing lager regions of homoeologous wheat genomes.

### Did accelerated gene transfer accompany polyploidization?

Although dating duplication events is difficult without the knowledge of donor and acceptor sequences, some estimates can be done. Choulet *et al.*[10] found that the most re-arranged regions of wheat chromosome 3B, including ctg0954b, exhibited most recent TE activity (1.0 – 1.4 million years on average), while regions with the oldest TE activity (1.9 – 2.1 MY) were most conserved. Hence, most of the insertions should be recent.

However, our result showed that two thirds of genes non-collinear with *Brachypodium* and rice are shared between the two wheat genomes. Thus the non-collinear genes had to be inserted before the divergence of all wheat genomes from a common ancestor about 2.5 – 4.5 MYA [42] or even before the divergence of the barley lineage 10 MYA [5] as some of the genes are conserved at the orthologous barley Rph7 locus [GenBank:AF521177] [10,43]. Thus, relocation of these genes cannot be linked with the recent TE activity. The remaining one third of non-collinear genes (seven and five in the 3DS and 3B loci, respectively) had to be inserted after the divergence of wheat B and D genome from a common ancestor about 2.5 – 4.5 MYA [42].

The fact the two thirds of genes were inserted during the 30 MY of evolution after the divergence of the *Brachypodium* and wheat lineages (35 MYA [44]) and that one third of the genes were inserted during the past 2.5 – 4.5 MY indicates a three- to five-fold higher rate of gene insertion after divergence of the wheat diploid progenitors. During this period, the only significant event, which could stimulate gene movement, was interspecific hybridization. Lai *et al.*[13] detected significant acceleration of gene movement and loss in the polyploid maize genome compared to its diploid progenitors. Using ancestral (1–2 million years old) and artificially generated *Gossypium* hybrids, Flagel *et al.*[45] estimated that ¼ of expression changes are related to allopolyploidy. Here, understanding gene movements, which accompany hybridization and polyploidization, is not possible without obtaining additional sequences from ancestors of wheat genomes as well as from the wheat genome itself.

### Less pseudogenes are found at the 3DS locus compared to 3B

Wicker *et al.*[11] argued that many of the non-collinear genes were either pseudogenes or gene fragments. Here, we found evidence for only two obvious pseudogenes (see Methods for definition) among the 39 coding sequences on 3DS locus (Taa3DS_ctg1484.00030 and Taa3DS_ctg1484.00050). Moreover, one of the pseudogenes is at a perfect collinear position in all four regions of the 3 genomes while the second one is a tandem duplication (see Figure 1). Interestingly, the 3B locus carries only one copy of this particular pseudogene (TAA_ctg0954b.00150) thereby indicating that the duplication took place after the loss of gene function and the divergence between the wheat genome progenitors. In contrast, six pseudogenes and gene fragments were identified among the same number of CDS on 3B. The first one is a homologue of Taa3DS_ctg1484.00030 and Taa3DS_ctg1484.00050 and hence is collinear with *Brachypodium* and rice. The remaining four have homologues on 3DS with intact ORF, while the last one is specific to the 3B locus. Thus, no pseudogene was found among the non-collinear genes at the 3DS locus whereas five out of six pseudogenes/gene fragments found on 3B are at non-collinear positions compared to *Brachypodium* and rice. The pseudogenes/gene fragments represent a small fraction of non-collinear genes (5/22) at the 3B locus. Altogether these results indicate that most if not all non-collinear genes present at the 3B and 3D orthologous loci do not display structural features of pseudogenes.

### Pseudogene formation after insertion

**Figure 2:**
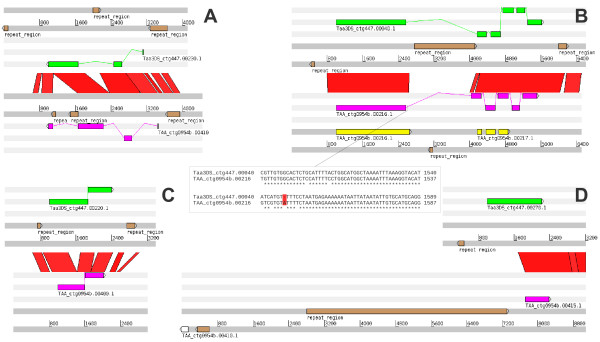
**Comparison of gene – pseudogene homoeologous pairs.** Intact coding sequences (all from the 3DS locus) are in upper trace highlighted in green colour. Pseudogenes (from 3B locus) are displayed in the lower trace in pink. Sequence features shown in brown represent repetitive elements. Red connections between traces represent Blastn hits between sequences. **A**) Insertion of repetitive element into the last exon of TAA_ctg0954b.00410 gene caused its pseudogenization. **B**) Pseudogene TAA_ctg0954b.00216 was originally annotated as two different gene fragments (yellow features). Comparison with the orthologous 3DS gene allowed their annotation as one pseudogene. Sequence alignment revealed insertion of single nucleotide causing frame shift (sequence cut-out). Remaining two genes TAA_ctg0954b.00400 ( **C**) and TAA_ctg0954b.00415 ( **D**) lost their function by partial deletion. While inner deletion disrupted gene TAA_ctg0954b.00400 ( **C**), 5' end of TAA_ctg0954b.00415 ( **D**) was removed. No homology with 3DS orthologous gene Taa3DS_ctg447.00270.1 was found upstream to closest gene (leftmost feature in white).

What is the origin of pseudogenes? Wicker *et al.*[11] proposed that many wheat pseudogenes arose from insertions of imperfect sequences, either through double-strand break repair or TE capture. However, four of the pseudogenes found at the 3B locus have homologs with complete ORF on the 3DS locus thereby suggesting pseudogene formation only after insertion. A detailed comparisons of four gene (3DS) – pseudogene (3B) pairs (Figure 2) revealed that one pseudogene (TAA_ctg0954b.00410) was formed by a TE insertion in one of the exons (Figure 2A) and that another one (TAA_ctg0954b.00216) arose from a frame-shift mutation caused by a single nucleotide insertion (Figure 2B). The two remaining CDS (TAA_ctg0954b.00400 and TAA_ctg0954b.00415) (Figure 2C and D) were pseudogenized after partial deletion of coding sequence.

These results suggest that pseudogenization occurred predominatly after the formation of allopolyploid wheat. First, genes are not under a strong selection after polyploidization. Second, all pseudogenes were found in 3B genome, and this can be explained by the longer time the B genome spent in a polyploid context as compared to the D genome. Pseudogene sequences can be eroded and lost over extended periods of time. Such gene loss was found in maize and *Arabidopsis* during the past five and ten million years following polyploidization events, respectively [12,13]. In our case, two of 3BS pseudogenes were not deleted but degraded to some extent after ~0.5 million years since the hybridization between *Triticum urartu* and *Aegilops spletoides*.

### Non-collinear genes carry promoter sequences and are likely expressed

The promoter region is essential to drive gene expression and many pseudogenes actually lack a promoter or have a non-functional sequence [46]. We searched for promoter-like sequences 1 kb upstream of the annotated CDS at the homoeologous 3DS and 3BS loci to provide further support to the conclusion that most of non-collinear CDS at the locus studied are not pseudogenes. Transcription start sites (TSS) were identified for 54.2% and 54.5% of non-collinear genes at 3DS and 3B locus, respectively (Additional file 4: Table S3). A TATA-box was found for a majority of them 14–38 bp upstream to the TSS. Among the collinear genes, TSSs were identified for about 73% of them. These findings indicate that not many non-collinear CDS lack a promoter sequence and hence a majority of them may be expressed. To date, only about 16,000 wheat full-length cDNAs are publicly available thereby limiting a possibility to identify fully expressed genes. As a result, only coding sequences could be annotated in the present work, while transcription start sites predicted by TSSP could not be compared to 5' ends of fl-cDNA.

Wheat ESTs publicly available in GenBank were then used to assess the expression of genes at the two homoeologous loci. Stringent criterion of 100% identity was used to score the number of ESTs matching each gene in the 3DS and 3B loci. Four ESTs hit homologous gene pairs and were removed from the analysis. 41.7% of the non-collinear genes at 3DS had at least one hit in the EST collection (Additional file 5: Table S4) indicating their expression. The ratio of expressed genes was slightly higher (53.3%) among the genes found at collinear position with *Brachypodium* and rice. Similarly, collinear genes at the 3B locus were more frequently expressed (33.3% vs. 22.7%). Interestingly, genes in the 3B locus seemed less expressed than genes at the homoeologous 3DS locus. We have performed two-tailed binomial test for each pair of homoeologous genes differing in expression. Unfortunately, 3DS genes have significantly higher level of expression than their 3B counterparts only in two cases. A similar reduction of non-collinear gene expression was found also for genes on wheat group 1 chromosomes (Wicker *et al.* 2011). As in case of higher fraction of pseudogenes at the 3B locus, the longer coexistence of the B genome in polyploid wheat genome as compared to the D genome may explain the reduced number of expressed genes.

## Conclusion

The availability of sufficient genome coverage and well assembled sequences is a prerequisite to study the evolutionary genome changes that accompanied speciation and domestication of cultivated plants. We have performed a direct comparison of two Mb-sized regions of the B and D genomes of bread wheat and revealed similar rates of non-collinear gene insertion in both genomes with a majority of gene duplications occurring before their divergence. Relatively low proportion of pseudogenes was identified among non-collinear coding sequences suggesting that pseudogenization did not accompany gene insertion events, but occurred later during the evolution of the polyploid genome. Full chromosome sequencing is underway under the umbrella of the IWGSC (wheatgenome.org) and the availability of complete reference chromosome sequences will enable confirming these findings and provide further insights into the mechanisms involved in shaping the polyploid wheat gene space. These observations will be supported by RNASeq studies to assess the relative expression of the homoeologous copies and relate structural and functional information.

## Competing interests

The authors declare that they have no competing interests.

## Authors’ contribution

JB, CV, PS, CF and JD designed the experiments. JS and HSi constructed the BAC library. JB, FCa and HB build the physical map and defined and reordered MTP. JS and KC screened the BAC library with molecular markers. CV and MD isolated DNA and sequenced BAC clones. CV, JP and HSt assembled the sequence. JB and FCh annotated the sequence. JB performed comparative analysis. JB and JD drafted manuscript and CF and PS edited the manuscript. All authors carefully read and approved the final manuscript.

## Supplementary Material

Additional file 1 **Table S1. **List of EST and PCR primers used for BAC library screening and identification of contigs from target region. Click here for file

Additional file 2 **Table S2. **Statistics of shotgun BAC clone sequencing and assemblies. Click here for file

Additional file 3 **Figure S1.** 454 reads mapped to assembled sequences of the two BAC contigs Click here for file

Additional file 4 **Table S3.** Promoter prediction for genes found at Hga locus at chromosome arm 3DS and at homoeologous region of chromosome 3B. Click here for file

Additional file 5 **Table S4.** Analysis of expression for genes found at Hga locus at chromosome arm 3DS and at homoeologous region of chromosome 3B. Click here for file
