# Supplementary material for: Intraspecific sequence comparisons reveal similar rates of non-collinear gene insertion in the B and D genomes of bread wheat
Source: BMC Plant Biol. 2012 Aug 30;12:155. doi: 10.1186/1471-2229-12-155 (PMC3445842; doi:10.1186/1471-2229-12-155)
Supplement: Additional file 1 — Table S1. List of EST and PCR primers used for BAC library screening and identification of contigs from target region. [file 1471-2229-12-155-S1.pdf]

Supplementary table 1. List of EST and PCR primers used for BAC library screening and identification of contigs from target region.

EST	Forward primer	Reverse primer
BE398268	GTGTAGATCTAAGGAAGGGCAGATT	GTATGTCTCCCACAGAAACTTGAAC
BE412128	ACAGTCATCGGCAAGATTCC	ACCCGGAATATCAATCACCA
BE427255	GTCCAATCCGGTCACTCATT	CAGTCCACTGCTCATCTGGT
BE499148	CAAGATCAAGAAGGCCAAGC	AGGTACACCCCGTTCTCGAT
BE606515	GAGGTGCAGTTCCTCGTCTC	GGTGAAGTCGACCATGGAGT
BF484268	CAAGATCAAGAAGGCCAAGC	AGGTACACCCCGTTCTCGAT
BJ222861	ACAAGAAGCCGAGGATGATG	AGGAAGAGCGAGTTGGTGAG
BJ227880	AGCGGCTACTCTCTGAGCAT	GGACATGGGGCTCAAGTACA
BJ303565	AGAAGGCACAGGAAACATGG	ACCCTTCGCTCATCATCATC
TC268917	TTCAGTAACCACGGAGCTTT	GAAAAAGGGAGGAGGTGTCC
TC57899	AGAAGAAGGAAGCCCCTCTG	GCCATGTCTTTTGTGCCTTT
TC57902	AGGAGCCGCTCTATGTCCTT	ATGCCAATCAGGTTCTGAGG
