# Supplementary material for: Intraspecific sequence comparisons reveal similar rates of non-collinear gene insertion in the B and D genomes of bread wheat
Source: BMC Plant Biol. 2012 Aug 30;12:155. doi: 10.1186/1471-2229-12-155 (PMC3445842; doi:10.1186/1471-2229-12-155)
Supplement: Additional file 2 — Table S2. Statistics of shotgun BAC clone sequencing and assemblies. [file 1471-2229-12-155-S2.pdf]

Supplementary table 2. Statistics of shotgun BAC clone sequencing and assemblies.

	BAC	No. Reads	No. Bases	Large contigs (>500) No. Contigs	No. Bases	N50
**A**	**cluster A**			**526**		
A01	45N17	33 917	11 298 470	28	117 086	6 029
A02	65O14	29 976	10 308 700	13	66 945	7 017
A03	08G17	6 687	1 872 659	22	109 237	8 457
A04	23I21	7 413	2 062 177	26	133 101	7 332
A05	72N17	39 219	13 157 697	24	129 276	8 466
A06	82H07	34 339	11 500 162	24	119 045	7 552
A07	72H10	30 531	10 411 681	14	122 386	11 682
A08	37C23	46 129	15 799 959	47	156 944	8 860
A09	31N24	34 949	11 127 314	43	132 425	8 808
A10	09G11	23 049	5 267 324	83	83 961	913
A11	04D04	18 230	5 953 506	29	147 842	7 999
A12	87O11	22 218	7 494 215	24	135 574	8 679
A13	58C08	19 305	6 503 956	17	109 904	9 526
A14	24N06	3 418	949 666	50	98 423	3 274
A15	11A13	7 979	2 231 454	15	106 637	13 617
A16	55O10	30 741	10 294 930	19	129 525	10 073
A17	15G21	7 485	2 103 329	18	110 492	8 368
A18	36F12	25 660	8 809 632	30	121 030	6 749
**B**	**cluster B**			**303**		
B01	52A05	16 440	5 427 047	36	113 208	4 757
B02	54D16	23 785	7 864 988	29	115 899	5 496
B03	53N07	33 238	10 972 361	59	165 918	7 759
B04	89C07	41 915	13 981 370	24	116 721	8 036
B05	90J06	29 625	9 989 963	155	295 326	6 539
**C**	**Singleton**					
C01	61C24	26 345	8 880 582	30	110 624	6 300
