# Supplementary material for: Intraspecific sequence comparisons reveal similar rates of non-collinear gene insertion in the B and D genomes of bread wheat
Source: BMC Plant Biol. 2012 Aug 30;12:155. doi: 10.1186/1471-2229-12-155 (PMC3445842; doi:10.1186/1471-2229-12-155)
Supplement: Additional file 3 — Figure S1. 454 reads mapped to assembled sequences of the two BAC contigs [file 1471-2229-12-155-S3.pdf]

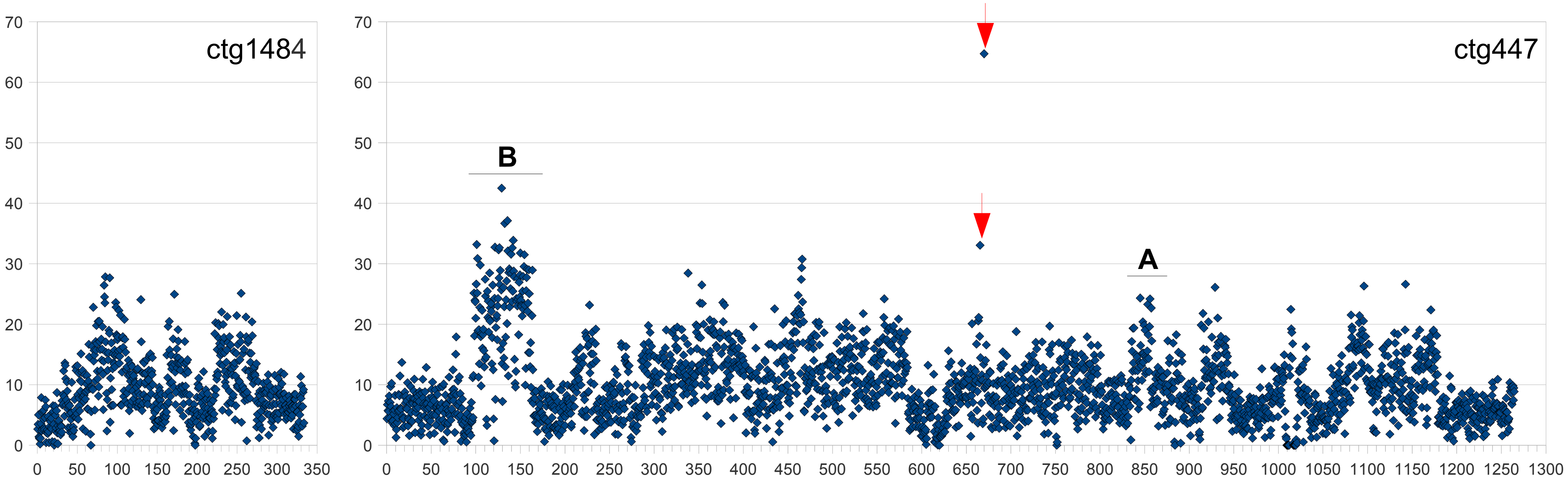

Supplementary figure 1. 454 reads mapped to assembled sequencies of the two BAC contigs. Reads obtained after sequencing 3-kb paired-end library were aligned to assembled scaffolds using Mosaik (http://code.google.com/p/mosaik-aligner/) and the read depth was calculated in 500 bp windows. Y-axis represents corresponding mean read depth and x-axis represents sequence in kb. Bias in read depth is expected primarily due to increased number of reads in the regions where neighbouring clones are overlapping (A; clones TaaCsp3DShA_0087O11 and TaaCsp3DShA_0058C08 overlap at 835 – 858 kb). Unequal amount BAC DNA pooled prior paired-end library construction may also contribute to the depth bias (B; clone TaaCsp3DShA_0065O14 at 97 – 164 kb). Red arrows indicate a single case we have identified, where repetitive DNA could increase read depth. LTR of RLG_Latidu_Taa3DS_ctg447-2 are highly over-represented among the 454 reads.
