# Supplementary material for: Intraspecific sequence comparisons reveal similar rates of non-collinear gene insertion in the B and D genomes of bread wheat
Source: BMC Plant Biol. 2012 Aug 30;12:155. doi: 10.1186/1471-2229-12-155 (PMC3445842; doi:10.1186/1471-2229-12-155)
Supplement: Additional file 4 — Table S3. Promoter prediction for genes found at Hga locus at chromosome arm 3DS and at homoeologous region of chromosome 3B. [file 1471-2229-12-155-S4.pdf]

Supplementary table 3. Promoter prediction for genes found at Hga locus at chromosome arm 3DS and at homoeologous region of chromosome 3B.‡

Non-collinear genes					
3DS	TSS to Start codon	TATA-box to TSS	3B	TSS to Start codon	TATA-box to TSS
Taa3DS_ctg1484.00020.1	797	17			
Taa3DS_ctg1484.00040.1					
Taa3DS_ctg1484.00050.1*					
Taa3DS_ctg447.00010.1			TAA_ctg0954b.00180.1	606	38
Taa3DS_ctg447.00020.1	468	38	TAA_ctg0954b.00200.1	445	35
			TAA_ctg0954b.00210.1		
Taa3DS_ctg447.00030.1	171	35	TAA_ctg0954b.00215.1	172	37
Taa3DS_ctg447.00040.1			TAA_ctg0954b.00216.1*		
Taa3DS_ctg447.00050.1	527	63	TAA_ctg0954b.00220.1	387	18
Taa3DS_ctg447.00070.1	728	28	TAA_ctg0954b.00240.1	352	
Taa3DS_ctg447.00090.1	133	38	TAA_ctg0954b.00260.1	172	13
			TAA_ctg0954b.00270.1	72	38
Taa3DS_ctg447.00100.1			TAA_ctg0954b.00280.1		
Taa3DS_ctg447.00120.1	120	28	TAA_ctg0954b.00300.1	126	28
Taa3DS_ctg447.00140.1			TAA_ctg0954b.00320.1		
Taa3DS_ctg447.00160.1			TAA_ctg0954b.00340.1	702	29
			TAA_ctg0954b.00350.1*		
Taa3DS_ctg447.00170.1			TAA_ctg0954b.00360.1	308	16
Taa3DS_ctg447.00190.1	75	14			
Taa3DS_ctg447.00200.1	54	35	TAA_ctg0954b.00380.1	54	35
Taa3DS_ctg447.00220.1			TAA_ctg0954b.00400.1*		
Taa3DS_ctg447.00230.1	116	36	TAA_ctg0954b.00410.1*		
Taa3DS_ctg447.00240.1	248	33			
Taa3DS_ctg447.00250.1					
Taa3DS_ctg447.00260.1	66				
Taa3DS_ctg447.00270.1			TAA_ctg0954b.00415.1*		
Taa3DS_ctg447.00320.1	326	37	TAA_ctg0954b.00460.1	211	33
			TAA_ctg0954b.00470.1		
			TAA_ctg0954b.00480.1		

Collinear genes					
**3DS**	**TSS to Start codon**	**TATA-box to TSS**	**3B**	**TSS to Start codon**	**TATA-box to TSS**
Taa3DS_ctg1484.00010.1			TAA_ctg0954b.00140.1	618	14
Taa3DS_ctg1484.00030.1*	853		TAA_ctg0954b.00150.1*		
Taa3DS_ctg1484.00060.1			TAA_ctg0954b.00160.1		
Taa3DS_ctg447.00060.1	604	14	TAA_ctg0954b.00230.1	659	29
Taa3DS_ctg447.00080.1	132	36	TAA_ctg0954b.00250.1	118	34
Taa3DS_ctg447.00110.1	229	37	TAA_ctg0954b.00290.1	590	30
Taa3DS_ctg447.00130.1			TAA_ctg0954b.00310.1		
Taa3DS_ctg447.00150.1	885	36	TAA_ctg0954b.00330.1	317	34
Taa3DS_ctg447.00180.1	130	32	TAA_ctg0954b.00370.1	51	
Taa3DS_ctg447.00210.1			TAA_ctg0954b.00390.1		
Taa3DS_ctg447.00280.1	380		TAA_ctg0954b.00420.1	606	37
Taa3DS_ctg447.00290.1	51		TAA_ctg0954b.00430.1	475	33
Taa3DS_ctg447.00300.1	155		TAA_ctg0954b.00440.1	112	
Taa3DS_ctg447.00310.1	110	35	TAA_ctg0954b.00450.1	101	35
Taa3DS_ctg447.00330.1	148	35	TAA_ctg0954b.00490.1	150	36

‡ Transcription start sites (TSS) and TATA-box sequence were identified upstream to start codon using TSSP (Shahmuradov *et al*, 2005). 1000 bp upstream to start codon were used for the analysis. Non-collinear genes specific for one genome are labelled red and genes shared between the homoeologous chromosomes 3D and 3B are labelled in blue. Pseudogenes and gene fragments are marked by an asterisk.
