# Supplementary material for: Intraspecific sequence comparisons reveal similar rates of non-collinear gene insertion in the B and D genomes of bread wheat
Source: BMC Plant Biol. 2012 Aug 30;12:155. doi: 10.1186/1471-2229-12-155 (PMC3445842; doi:10.1186/1471-2229-12-155)
Supplement: Additional file 5 — Table S4. Analysis of expression for genes found at Hga locus at chromosome arm 3DS and at homoeologous region of chromosome 3B. [file 1471-2229-12-155-S5.pdf]

Supplementary table 4. Analysis of expression for genes found at Hga locus at chromosome arm 3DS and at homoeologous region of chromosome 3B.‡

Non-collinear genes				
**3DS**	**3DS_matching_ESTs**	**3B**	**3B_matching_ESTs**	**P-values of two-tailed binomial test**
Taa3DS_ctg1484.00020.1				
Taa3DS_ctg1484.00040.1	1			
Taa3DS_ctg1484.00050.1*				
Taa3DS_ctg447.00010.1	3	TAA_ctg0954b.00180.1	1	0,63
Taa3DS_ctg447.00020.1	26	TAA_ctg0954b.00200.1	9	**0,006**
		TAA_ctg0954b.00210.1		
Taa3DS_ctg447.00030.1	3	TAA_ctg0954b.00215.1	3	
Taa3DS_ctg447.00040.1		TAA_ctg0954b.00216.1*		
Taa3DS_ctg447.00050.1	1	TAA_ctg0954b.00220.1	1	
Taa3DS_ctg447.00070.1	2	TAA_ctg0954b.00240.1	0	0,5
Taa3DS_ctg447.00090.1	3	TAA_ctg0954b.00260.1	0	0,25
		TAA_ctg0954b.00270.1		
Taa3DS_ctg447.00100.1		TAA_ctg0954b.00280.1		
Taa3DS_ctg447.00120.1		TAA_ctg0954b.00300.1		
Taa3DS_ctg447.00140.1	0	TAA_ctg0954b.00320.1	2	0,5
Taa3DS_ctg447.00160.1		TAA_ctg0954b.00340.1		
		TAA_ctg0954b.00350.1*		
Taa3DS_ctg447.00170.1	0	TAA_ctg0954b.00360.1	1	0,5
Taa3DS_ctg447.00190.1	1			
Taa3DS_ctg447.00200.1		TAA_ctg0954b.00380.1		
Taa3DS_ctg447.00220.1		TAA_ctg0954b.00400.1*		
Taa3DS_ctg447.00230.1		TAA_ctg0954b.00410.1*		
Taa3DS_ctg447.00240.1	2			
Taa3DS_ctg447.00250.1				
Taa3DS_ctg447.00260.1	1			
Taa3DS_ctg447.00270.1	2	TAA_ctg0954b.00415.1*	0	0,5
Taa3DS_ctg447.00320.1		TAA_ctg0954b.00460.1		
		TAA_ctg0954b.00470.1		
		TAA_ctg0954b.00480.1		

Collinear genes				
**3DS**	**3DS_matching_ESTs**	**3B**	**3B_matching_ESTs**	**P-values of two-tailed binomial test**
Taa3DS_ctg1484.00010.1	1	TAA_ctg0954b.00140.1	1	
Taa3DS_ctg1484.00030.1*		TAA_ctg0954b.00150.1*		
Taa3DS_ctg1484.00060.1	9	TAA_ctg0954b.00160.1	1	**0,02**
Taa3DS_ctg447.00060.1		TAA_ctg0954b.00230.1		
Taa3DS_ctg447.00080.1	2	TAA_ctg0954b.00250.1	1	1
Taa3DS_ctg447.00110.1	0	TAA_ctg0954b.00290.1	3	0,25
Taa3DS_ctg447.00130.1		TAA_ctg0954b.00310.1		
Taa3DS_ctg447.00150.1		TAA_ctg0954b.00330.1		
Taa3DS_ctg447.00180.1		TAA_ctg0954b.00370.1		
Taa3DS_ctg447.00210.1	24	TAA_ctg0954b.00390.1	28	0,58
Taa3DS_ctg447.00280.1	24	TAA_ctg0954b.00420.1	20	0,65
Taa3DS_ctg447.00290.1		TAA_ctg0954b.00430.1		
Taa3DS_ctg447.00300.1	4	TAA_ctg0954b.00440.1	0	0,13
Taa3DS_ctg447.00310.1	1	TAA_ctg0954b.00450.1	0	0,5
Taa3DS_ctg447.00330.1	5	TAA_ctg0954b.00490.1	0	0,06

‡ 1,071,335 ESTs publicly available in GenBank were used for expression analysis of CDS. Standalone Blastn programme was used to perform a similarity search with following parameters: reward for a nucleotide match 2; expectation value $1e^{-10}$; and default settings for all other parameters. Only blast hits with 100% identity and minimal length of 100 bp were considered. The number of unique ESTs for each is gene is given in the table. Non-collinear genes specific for one genome are labelled red and genes shared between the homoeologous chromosomes 3D and 3B are labelled in blue. Pseudogenes and gene fragments are marked by an asterisk. P-values of two-tailed binomial test were calculated for each pair of genes with differential expression. Significant differences are labelled in red.
